# P-243. Catheter-Associated Urinary Tract Infections: What’s the secret?

**DOI:** 10.1093/ofid/ofae631.447

**Published:** 2025-01-29

**Authors:** Rawad Abi Assaad, Julian Maamari, Abdallah Mughrabi, Jorge Fleisher

**Affiliations:** St. Elizabeth's Medical Center - Boston University School of Medicine, Boston, Massachusetts; St. Elizabeth's Medical Center - Boston University School of Medicine, Boston, Massachusetts; St. Elizabeth's Medical Center - Boston University School of Medicine, Boston, Massachusetts; St. Elizabeth Medical Center, Boston, Massachusetts

## Abstract

**Background:**

In the United States, more than 1 million CAUTIs are diagnosed each year, ranking among the top 5 most common healthcare-associated infections; however, our observations suggest that these numbers are inflated due to the lack of clear diagnostic guidelines. Most laboratories process urine cultures (UC) in reflex when a urinalysis (UA) sample shows 10 or more cells/µL. By adding specific criteria to decrease the number of UC, we decreased unnecessary use of antibiotics and overall cost from CAUTI misdiagnosis.

**Methods:**

In this study, we enforced conditions that increase standards for reflex UC processing. These conditions included patient symptoms, presence of indwelling catheter, and levels of red blood and epithelial cells. We prospectively evaluated outcomes for patients whose UA samples did not meet our criteria. We suppressed UA samples that did not meet our criteria and did not report the culture results.

**Results:**

Analysis of UC requests from 2020 to 2023 revealed several notable findings: 37% of the urine samples were approved for culture, 44% of those yielded positive results, and 56% showed either mixed flora or no bacterial growth. All UC samples that met criteria had white blood cell counts between 25 and 50 /HPF, with 91% displaying over 50 /HPF. Notably, none of the excluded samples resulted in patients developing urinary complications.

Amongst the suppressed UC, 9% yielded positive culture results consistent with asymptomatic bacteriuria. We noticed CAUTI reductions from 31/year in 2018 to 8/year in 2023. Our intervention helped avoid unnecessary antibiotics use, decreasing the number of hospitals acquired Clostrioides difficile infections from 31 cases in 2018 to 9 cases in 2023.

**Conclusion:**

The high rejection rate of UC samples suggests opportunities for optimizing appropriateness criteria to improve diagnostic yield and resource utilization. Further analysis of rejection reasons may help identify areas for clinician education regarding appropriate UC ordering.

**Disclosures:**

**All Authors**: No reported disclosures

